# Validation and enhancement of a vocal fold medial surface 3D reconstruction approach for in-vivo application

**DOI:** 10.1038/s41598-023-36022-6

**Published:** 2023-07-03

**Authors:** Patrick Schlegel, Michael Döllinger, Neha K. Reddy, Zhaoyan Zhang, Dinesh K. Chhetri

**Affiliations:** 1 Department of Head and Neck Surgery, University of California, Los Angeles, UCLA Rehabilitation Services, 1000 Veteran Ave, Los Angeles, CA 90095 USA; 2 Department of Head and Neck Surgery, Division of Phoniatrics and Pediatric Audiology, Friedrich Alexander University Erlangen-Nürnberg, Erlangen, Germany

**Keywords:** Computational science, Biomedical engineering

## Abstract

In laryngeal research, studying the vertical vocal fold oscillation component is often disregarded. However, vocal fold oscillation by its nature is a three-dimensional process. In the past, we have developed an in-vivo experimental protocol to reconstruct the full, three-dimensional vocal fold vibration. The goal of this study is to validate this 3D reconstruction method. We present an in-vivo canine hemilarynx setup using high-speed video recording and a right-angle prism for 3D reconstruction of vocal fold medial surface vibrations. The 3D surface is reconstructed from the split image provided by the prism. For validation, reconstruction error was calculated for objects located at a distance of up to 15 mm away from the prism. The influence of camera angle, changing calibrated volume, and calibration errors were determined. Overall average 3D reconstruction error is low and does not exceed 0.12 mm at 5 mm distance from the prism. Influence of a moderate (5°) and large (10°) deviation in camera angle led to a slight increase in error to 0.16 mm and 0.17 mm, respectively. This procedure is robust towards changes in calibration volume and small calibration errors. This makes this 3D reconstruction approach a useful tool for the reconstruction of accessible and moving tissue surfaces.

## Introduction

The audible human voice is produced in the larynx, as the airstream rising from the lungs excites the vocal fold into vibration, which again modulates the airflow into sound waves, generating the fundamental frequency of voice^1^. Even though this voice production process is rather easily described in rough outlines, it has many complexities as it can be modulated in various ways and can be affected by various factors such as level of neuromuscular stimulation, tissue elasticity, subglottal pressure, etc.^2,3^. To achieve a better understanding of the control of voice production, many input and output signals are of interest, such as laryngeal nerve activation, changes in flow and pressure, or the produced acoustic signal. To better understand this complex process, ideally, an experimental setup is needed that can control or at least capture all relevant factors simultaneously and employs robust algorithms that do not introduce further confounding factors.

The vocal folds are two opposing structures in the larynx that form a constriction of the airway. They stretch across the larynx in an anterior–posterior direction and their exact posturing can be adjusted by the movement of various cartilages in the larynx, bringing them in close proximity for phonation^4^. As they are set in motion by the airstream rising from the lungs they oscillate with on average 235 Hz in women and 134 Hz in men during sustained vowel phonation, the sound source of voice^5^. This sound source is then further modulated by the acoustic resonances of the vocal tract, generating speech^4^.

A healthy voice is usually characterized by periodic and left–right symmetric oscillations of the vocal folds that close completely or almost completely in every cycle^6–8^. In contrast, phonatory disease is often characterized by aperiodic or asymmetric oscillations of the vocal folds and incomplete glottal closure during phonation^9–11^. Respectively many different measures are in use that try to capture the properties of vocal fold oscillations such as duration and ratios of opening, closing, and closed phases of the glottal opening area waveform^1,12^.

The medial surface of the vocal folds (i.e. the surface where the vocal folds meet during oscillation) is a critical vibratory area influencing acoustics^13^ and hence of special interest. Vocal folds do not simply close at the same time everywhere along this surface but rather display a pattern of closing from the bottom upwards and opening in the same way. Changes in this pattern, controlled by laryngeal muscle activations, can have a large influence on the resulting acoustic signal^13,14^.

However, in voice research, in-vivo experiments typically record the oscillating vocal folds from above, capturing vibration from a superior view in 2D^15^ or 3D^16^, but are unable to record the entire medial surface, nor collect data regarding muscle activation or medial surface dynamics^17–19^. The ex vivo hemilarynx allows visualization of the medial surface, but often no nerve activation can be achieved^20,21^ without applying special measures such as using perfused models^22^.

For in-vivo 3D reconstruction of biological tissue, several methods exist. One of the least invasive ones is high-resolution computed tomography which was applied for the 3D reconstruction of vocal folds^23^. However, the spatial resolution of this technique is limited with even modern ultra-high-resolution approaches only reaching a slice thickness of 0.25 mm^24^. Theoretically, higher accuracy can be achieved by direct high-speed video recording of the vocal folds from above in combination with laser projection or similar techniques. However, the accuracy of these approaches is confined by the spatial restrictions of the pharynx which currently limits vertical resolution to a value of about 0.36 mm^16^. Further, these techniques are confined to a superior view of the vocal folds and cannot fully visualize the complete medial surface^25^. Therefore, the only feasible way to accomplish resolutions on the sub 0.1 mm scale of the medial surface at present is the usage of an in-vivo hemilarynx for direct visualization.

The hemilarynx behaves similarly to a full larynx during phonation in regards to many important variables such as phonation threshold pressure, fundamental frequency, or vibrational amplitude^26^ that lead to its well-established usage in voice research^27–29^. The presented hemilarynx setup in this work also allows for direct stimulation of laryngeal nerves adjusting the remaining vocal fold of the hemilarynx and changing its vibration properties which would not be feasible in an ex-vivo model^20^. For this study mainly two types of laryngeal nerves are of interest: The recurrent laryngeal nerve (RLN) innervates all laryngeal intrinsic adductor muscles (i.e. close the glottal gap and stiffens the body layer) and the superior laryngeal nerve (SLN) innervates the cricothyroid muscles (CT) (i.e. elongate and tense the vocal folds)^4^. As experiments are conducted on a hemilarynx only one (here the left) vocal fold and respectively the left branches of these nerves remain.

Commercial tools for 3D surface reconstruction exist^30^ but have various limitations. They are closed source (which makes them opaque), managed externally (which prevents fast and efficient fixing of software-based errors), and expensive. Further, application of such tools that are developed with the intend of being applicable on a preferably wide array of problems on a very specific problem, like reconstruction of the vibrating vocal fold medial surface in-vivo, can result in poor performance.

To circumvent these problems, this work builds on a previous approach for 3D reconstruction of the oscillating hemi-larynx medial surface by Döllinger et al.^31,32^. In this previous work, the oscillating medial surface was recorded with high-speed video. For calibration, a brass cube of known dimensions fixed above the medial surface was used. Both, the medial surface and brass cube, were recorded through a right-angle transparent glass prism that generated a split view of the surface and cube at the same time. Using the known dimensions of the brass cube the surface could be reconstructed. However, this approach had three disadvantages: (1) as the brass cube occupied necessarily a different area than the medial surface, 3D reconstruction was based on considerable extrapolation of reconstructed space. (2) The brass cube only offered a small set of known 2 × 2D/3D position pairs for calibration (a set of seven visible cube vertices) (3) The brass cube and the medial surface had to be visible at the same time, hence only a smaller camera image section could be reserved for the medial surface.

In this work we present an updated version of this 3D reconstruction approach^31,32^, resolving the disadvantages of the previous process and enhancing the 3D reconstruction algorithm itself. For this purpose, we also build upon an already established technique for in-vivo laryngeal nerve stimulation as described by Chhetri et al.^2,3^. Both approaches are described in their relevant details in the methods section along with the changes we introduced to combine them and adapt them for 3D in-vivo hemilarynx surface reconstruction.

The aims of this validation study are as follows: (1) Propose an adapted setup and improved algorithm for 3D reconstruction of accessible moving tissue surfaces that are robust to changes in camera settings. (2) Validate this setup concerning various influencing factors such as distance of the reconstructed object, camera angle, and differences between calibration and reconstruction camera parameters. (3) Give an application example of the setup based on the 3D reconstruction of an in-vivo hemilarynx recording of the vibrating vocal fold medial surface with concurrent laryngeal nerve stimulation. (4) By making the source code available, we give colleagues the opportunity to use all or part of our code for their own specific 3D reconstruction tasks.

## Methods

### In vivo hemilarynx recording

**Figure 1:**
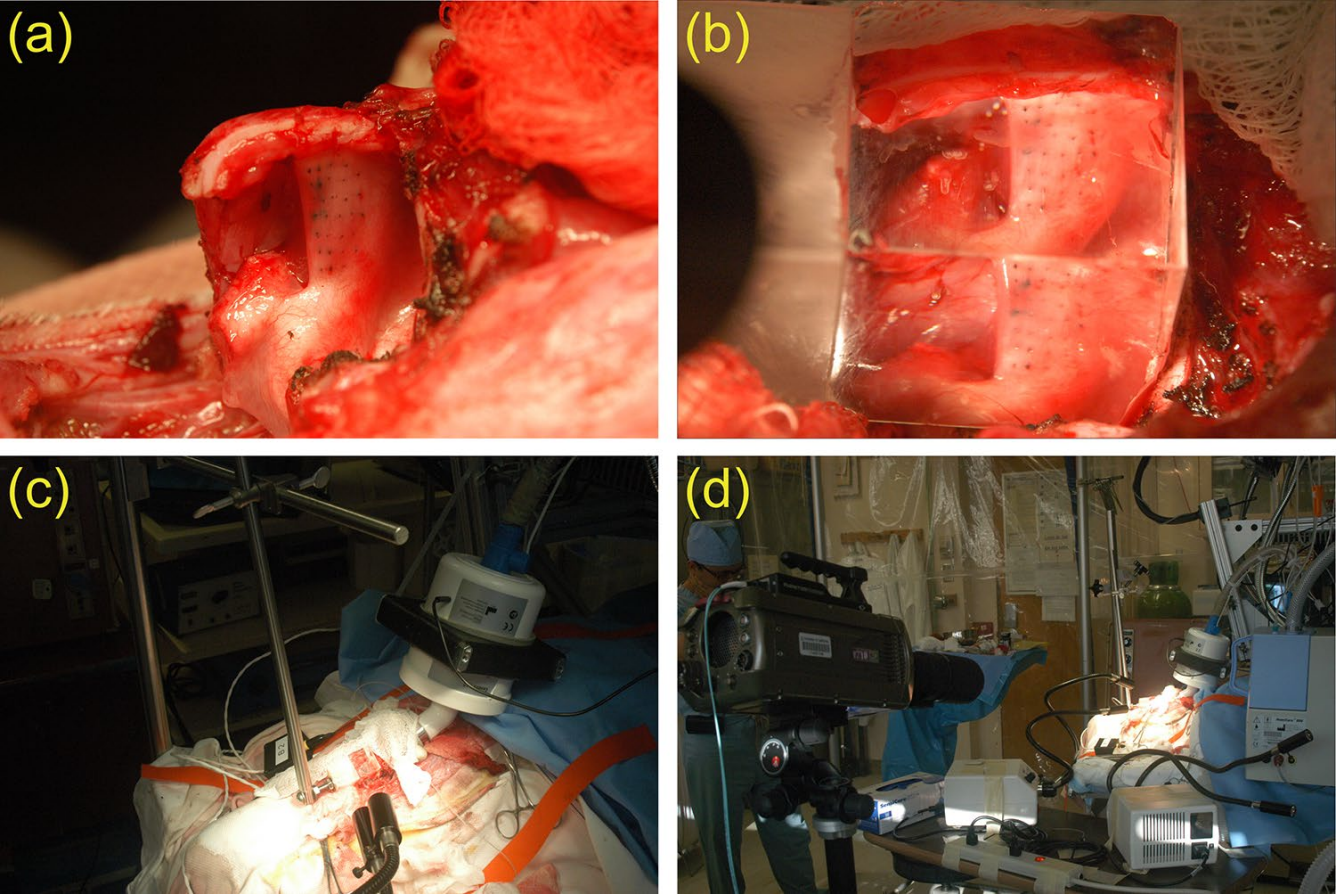
Surgical procedure to prepare the hemilarynx for recording: (**a**) Grid of India ink landmarks tattooed on the medial surface of the remaining vocal fold on the hemilarynx, (**b**) prism placed in front of the vocal fold providing a split view of the medial surface, (**c**) metal pole setup holding the prism in place, (**d**) high-speed video camera for the recording of vocal fold oscillations through the glass prism.

Data from one healthy male mongrel canine were used in this study for demonstration purposes. The data were originally collected for a previous study^14^. The performed surgical procedure to expose and prepare the hemilarynx was already described previously in detail in^2,3^ and hence will only be briefly summarized here with emphasis placed on details relevant to the 3D reconstruction algorithm:

The canine was anesthetized using isoflurane general endotracheal anesthesia and placed in a supine position on an operating table and ventilation was provided via a low tracheotomy. To provide controlled humidified airflow, a subglottic tube was attached to tracheal rings two and three, and an airflow controller (MCS Series Mass Flow Controller; Alicat Scientific, Tucson, AZ). The larynx was exposed and a right hemilaryngectomy was performed resulting in an in vivo hemilarynx with only the left vocal fold remaining. Figure 1a Using India ink a grid of 35 landmarks were tattooed on the medial surface of the vocal fold. Figure 1b A right-angle transparent glass prism (48 mm base, 35 mm length, 24 mm height) was placed against the vocal fold with the base on the hypotenuse side facing the medial surface. Figure 1c The prism was fixed tightly to the table by a setup of metal poles, ensuring a stationary position of the prism during the recording process. The two bases of the prism on the leg sides then provide a split view of the medial surface from two different perspectives. Figure 1d A Phantom v210 high-speed camera (Vision Research Inc., Wayne, NJ) was placed perpendicular to the prism, capturing both views.

**Figure 2:**
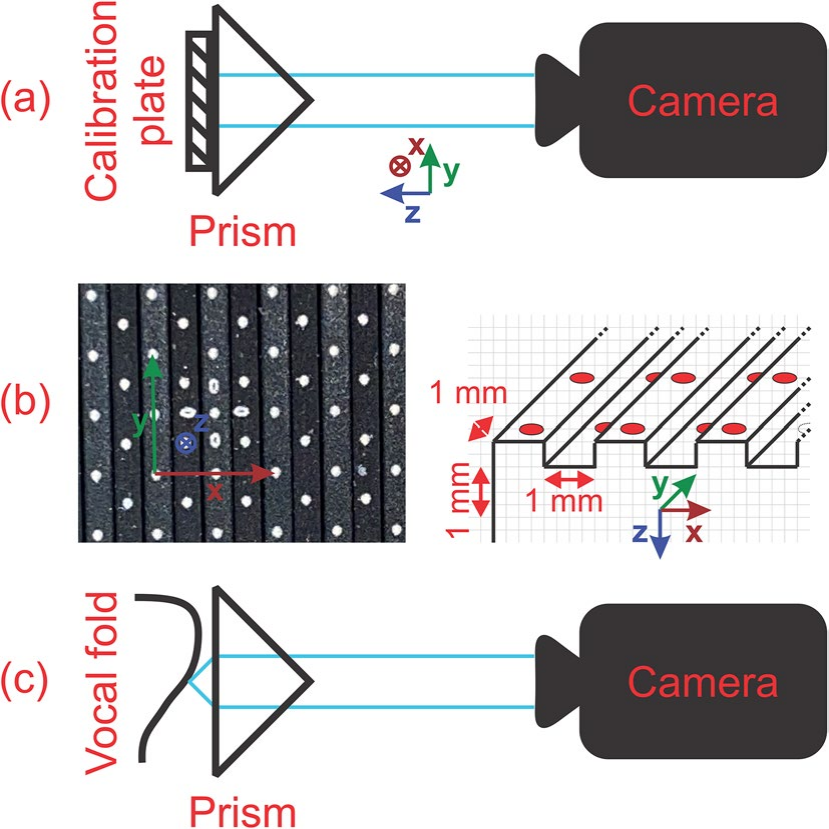
Visualization of (**a**) the 3D reconstruction setup for calibration, (**b**) calibration plate features and (**c**) setup for 3D reconstruction of the vocal fold medial surface with same camera parameters as used for calibration.

For calibration of the setup, a narrow calibration plate was inserted between the vocal fold and glass prism (dimensions 25 × 25 × 6 mm with 0.5 mm deep grooves). The plate was placed tight against the prism and one image of the plate was recorded with the camera. In Fig. 2a a schema of the recording setup is depicted. The calibration plate features multiple landmarks with known distances from each other in three dimensions as illustrated in Fig. 2b. These distances can then be used to map and extrapolate the reconstructed 3D space.

Subsequently, the plate was removed, and the medial surface of the vocal fold was recorded. By providing humidified airflow through the subglottic tube (at 1200 ml/s) and simultaneous stimulation of laryngeal nerves (see section “Phonatory neuromuscular stimulation”) phonation was achieved with the vocal fold oscillating against the glass prism. Oscillations of the vocal fold were recorded at 3000 frames per second for 1.5 s with a spatial resolution of 512 × 512 pixels. Just like the prism, the camera remained fixed during calibration and the entire recording process (see Fig. 2c). To verify that camera parameters remained constant (i.e. camera and prism position) the exact prism position in the recorded images is kept in view and a new calibration recording is done if necessary.

### Phonatory neuromuscular stimulation

To apply nerve stimulation during oscillation, superior and recurrent laryngeal nerves (SLN and RLN) of the left vocal fold were exposed and cuff electrodes (Ardiem Medical, Indiana, PA) were fixed to all nerves. In this way, cricothyroid muscle (CT) innervated by SLN, and laryngeal adductor muscles innervated by the RLN branch could be stimulated separately. By dividing the nerve branch innervating the posterior cricoarytenoid muscle, cross-stimulation was avoided.

For stimulation, 30 ms charge-balanced, biphasic cathodic pulses at 100 Hz were used throughout 1.5 s for each nerve. Eleven levels of SLN and RLN activation were tested for each nerve from no stimulation (0) to maximum stimulation (10). The effects of this stimulation were discussed in more detail by Reddy et al.^14^. However, this work does not focus on the effects of activation combinations, therefore only three activation conditions covering average stimulation levels ((1) RLN 4 SLN 5, (2) RLN 5 SLN 5, (3) RLN 6 SLN 5) were included for demonstrative purposes and basic parameters were calculated (see Surface interpolation and cycle detection).

### 3D reconstruction algorithm

The process of 3D reconstruction using two views is based on^33^ and can be expressed in a simplified manner using Eq. (1). In the first step (the calibration) known combinations of 2D points from both views in the image space and their resulting 3D positions in real space are used to get a preferably good approximation of the transformation function *T*. In a second step (the reconstruction) this function *T* then can be applied to new point pairs in image space to get their corresponding unknown 3D positions. Therefore, camera parameters (i.e. camera and prism positioning and angle) must stay the same during both steps.

1 $$\begin{aligned} & {\varvec{T}}: R^{2} \times R^{2} \to R^{3} \\ & {\varvec{T}}\left( {\left( {\begin{array}{*{20}c} {x`} \\ {y`} \\ \end{array} } \right) ,\left( {\begin{array}{*{20}c} {x^{\prime}} \\ {y^{\prime}} \\ \end{array} } \right) } \right) = \left( { \begin{array}{*{20}c} x \\ y \\ z \\ \end{array} } \right) | {\varvec{T}} = \user2{ }? \\ & T\left( {\left( {\begin{array}{*{20}c} {x^{A} } \\ {y^{A} } \\ \end{array} } \right),\left( {\begin{array}{*{20}c} {x^{B} } \\ {y^{B} } \\ \end{array} } \right)} \right) = \left. {\user2{ }\left( {\begin{array}{*{20}c} {{\varvec{x}}^{\user2{*}} } \\ {{\varvec{y}}^{\user2{*}} } \\ {{\varvec{z}}^{\user2{*}} } \\ \end{array} } \right)} \right| \left( {\user2{ }\begin{array}{*{20}c} {{\varvec{x}}^{\user2{*}} } \\ {{\varvec{y}}^{\user2{*}} } \\ {{\varvec{z}}^{\user2{*}} } \\ \end{array} } \right) = \user2{ }? \\ \end{aligned}$$

For calibration, at first, a grid of 17 × 9 points was marked on the calibration plate recordings using a custom software tool (GLabel, Friedrich Alexander University Erlangen-Nürnberg), thus providing an array of image space 2 × 2D point position pairs. Due to the known dimensions of and distances between points on the plate, a corresponding array of ideal real-space 3D positions for all points was defined.

A first estimated T* was obtained by applying the 2 × 2D to 3D mapping approach based on the linear approximation method described by Döllinger et al.^31^. This approach makes use of a calibration object that acts as an orthogonal basis in real space. In the experiment of Döllinger et al., a calibration cube was used that was mounted above the vocal fold and attached to the top of the prism. Therefore, the calibration object and vocal fold surface were always visible at the same time, but also not in the same space and the reconstruction was entirely dependent on extrapolation of the calibrated area. Using our setup, the orthogonal basis was derived from points on the calibration plate. This yields three advantages over the previous approach: (1) as the grid is placed in the same space as the vocal fold (see Fig. 2) x and y dimensions do not have to be extrapolated anymore (2) For the calibration of all dimensions not only the edges of a single cube but the entire grid, representing multiple “virtual” cuboids, can be used yielding more points for calibration and averaging. (3) A larger camera image section can be reserved for the medial surface, as the surface and calibration object do not have to be visible at the same time.

**Figure 3:**
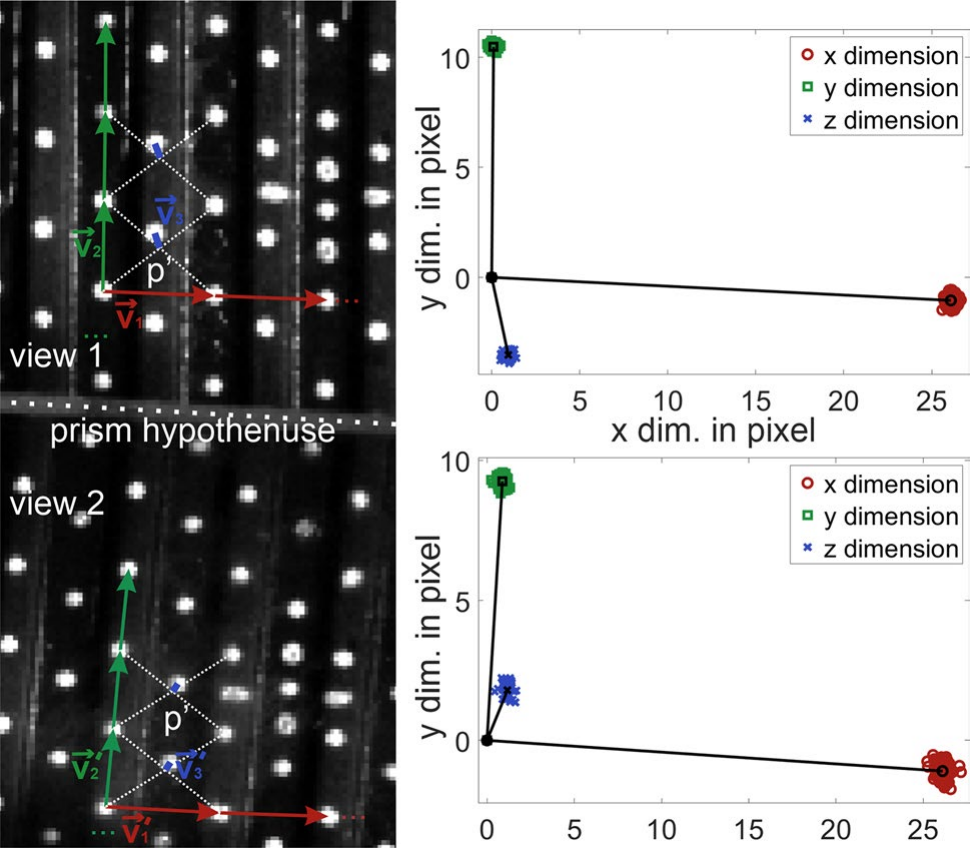
Reconstruction of an array of virtual cubes from calibration plate points.

In image space, virtual basis vectors ($\vec{v}_{1} ,\vec{v}_{2} ,\vec{v}_{3} \,{\text{and}}\, \vec{v}_{1}^{^{\prime}} ,\vec{v}_{2}^{^{\prime}} ,\vec{v}_{3}^{^{\prime}} \in R^{2}$) were reconstructed from the marked points for each view as shown in Fig. 3. Vectors in x- and y-direction were constructed by subtracting neighboring points on the grid. However, as no points on the grid were located “exactly above” one another in the z-direction (see Fig. 2b) the $\vec{v}_{3}$-vectors (as 2D representations of vectors that would point in z-direction in 3D space) had to be constructed indirectly. As depicted in Fig. 3 four points neighboring one point in a gauge were used to construct a virtual point p’ located exactly “above” the marked point in the gauge. Connecting these points, vectors in $\vec{v}_{3}$-direction could be constructed. The vectors were then averaged for each view and direction, resulting in three 2D vectors for each view ($\vec{v}_{1} = \left( {x_{1} ,y_{1} } \right), \vec{v}_{2} = \left( {x_{2} ,y_{2} } \right),\vec{v}_{3} = \left( {x_{3} ,y_{3} } \right)$ and $\vec{v}_{1} ^{\prime} = \left( {x_{1} ^{\prime},y_{1} ^{\prime}} \right), \vec{v}_{2} ^{\prime} = \left( {x_{2} ^{\prime},y_{2} ^{\prime}} \right),\vec{v}_{3} ^{\prime} = \left( {x_{3} ^{\prime},y_{3} ^{\prime}} \right)$).

Subsequently, matrix $F$, describing the linear mapping from 3D + 1 real space to 2 × 2D image space ($F: R^{4} \to R^{2} \times R^{2}$), was constructed from the basis vectors $\vec{v}_{1} ,\vec{v}_{2} ,\vec{v}_{3} and \vec{v}_{1}^{^{\prime}} ,\vec{v}_{2}^{^{\prime}} ,\vec{v}_{3}^{^{\prime}} \in R^{2}$ with an added fourth basis vector as a “helper dimension” to enable matrix inversion as shown in Eq. (2). Using Nelder-Mead optimization the first optimized $F^{*}$ was computed and the inversion of $F^{*}$ can be used for mapping the 2 × 2D image space to 3D + 1 real space. Therefore, the 3D reconstruction algorithm as proposed by Döllinger et al. was at this point reproduced^31^.

2 $$F^{*} = \left( {\begin{array}{*{20}c} {x_{1} } & {x_{2} } & {x_{3} } & 0 \\ {y_{1} } & {y_{2} } & {y_{3} } & 0 \\ {x_{1} ^{\prime}} & {x_{2} ^{\prime}} & {x_{3} ^{\prime}} & 0 \\ {y_{1} ^{\prime}} & {y_{2} ^{\prime}} & {y_{3} ^{\prime}} & {y_{4} ^{\prime}} \\ \end{array} } \right), \,\,F^{* - 1} \cdot \left( {\begin{array}{*{20}c} {x`} \\ {\begin{array}{*{20}c} {y`} \\ {x^{\prime}} \\ \end{array} } \\ {y^{\prime}} \\ \end{array} } \right) = \left. {\left( {\user2{ }\begin{array}{*{20}c} {{\varvec{x}}^{\# } } \\ {{\varvec{y}}^{\# } } \\ {{\varvec{z}}^{\# } } \\ \end{array} } \right) } \right| \left( {\user2{ }\begin{array}{*{20}c} {{\varvec{x}}^{\# } } \\ {{\varvec{y}}^{\# } } \\ {{\varvec{z}}^{\# } } \\ \end{array} } \right) = p^{\# }$$

To align the reconstructed 3D points $p^{\# }$ with a chosen useful real space coordinate system a certain rotation and shift must be applied to the reconstructed points. A rotation Matrix R and shift vector S were determined aligning reconstructed 3D grid points $p^{\# }$ to their real space 3D positions defined by the dimensions of the calibration plate. If the initial estimation $F^{*}$ does not yield a root mean square error between the reconstructed 3D positions $p^{\# }$ and the ideal real space 3D positions of less than 0.08 mm, further iterative Nelder-Mead optimization takes place minimizing the quadratic error sum, optimizing F, R, and S in the process until the algorithm yields no further improvement within 100 iterations. This allows for a successful 3D reconstruction even in cases where the original algorithm would have resulted in a warped image (e.g. if the angle between the camera and prism is too high). Therefore, the final Transformation function *T* is shown in Eq. (3). The entire algorithm was implemented in Matlab (R2021b) Update 1).

3 $$T\left( {\left( {\begin{array}{*{20}c} {x^{A} } \\ {y^{A} } \\ \end{array} } \right),\left( {\begin{array}{*{20}c} {x^{B} } \\ {y^{B} } \\ \end{array} } \right)} \right) = R\left[ { F^{ - 1} \cdot \left( {\begin{array}{*{20}c} {x^{A} } \\ {\begin{array}{*{20}c} {y^{A} } \\ {x^{B} } \\ \end{array} } \\ {y^{B} } \\ \end{array} } \right)} \right] + S = \left( {\user2{ }\begin{array}{*{20}c} {{\varvec{x}}^{\user2{*}} } \\ {{\varvec{y}}^{\user2{*}} } \\ {{\varvec{z}}^{\user2{*}} } \\ \end{array} } \right)$$

### Reconstruction validation

**Figure 4:**
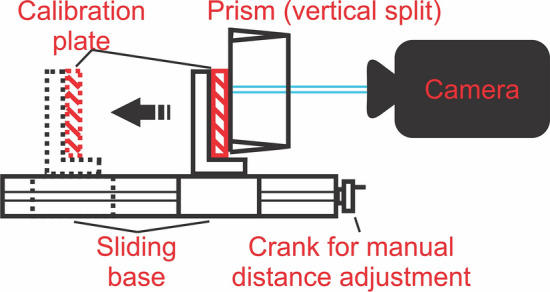
Illustration of the setup used for validation of the 3D reconstruction algorithm.

By comparing the reconstructed 3D coordinates with the optimal theoretical positions only exact information about the error with respect to the 3D space covered by the calibration plate can be obtained, i.e. a volume that will later cover the whole vocal fold in x and y dimension but only covers 0.5 mm in z-direction (from and towards the glass prism surface). It is expected that the error will slowly increase with increasing distance from the glass prism due to the underlying linear approximation method extrapolating 3D positions and a decreasing accuracy of marked points with increasing distance to the glass. For this reason, the following experiment was designed to analyze how the error behaves up to 15 mm from the glass surface:

A manual positioning system (A40 Series UniSlide, Velmex Inc., Bloomfield, NJ) was set up and the right-angle transparent glass prism was fixed in position above the anterior end of the system. For reasons of practicability, the prism was fixed for a vertical split of the views. The calibration plate was placed on the anterior end of the system in an upright position, in contact with the base on the hypotenuse side of the prism. Using the stationary Phantom v210 high-speed camera multiple images were recorded of the plate being moved away from the prism in 15 1 mm steps from 0 mm from the prism surface up to a distance of 15 mm. Distance adjustment was done manually with an accuracy of 0.01 mm. The distance between the camera and the prism surface was 1 m which corresponds to the upper limit distance that is used in the in-vivo experiments where the camera is often placed closer. The entire setup is schematically illustrated in Fig. 4.

**Figure 5:**
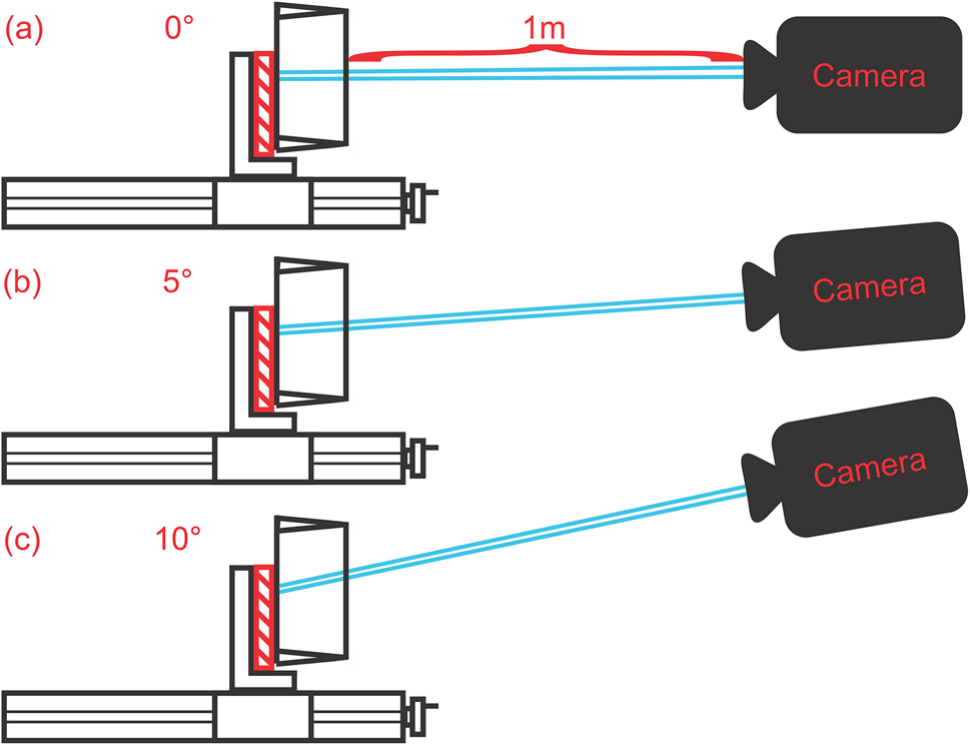
Illustration of the transversal axis rotations by (**a**) 0°, (**b**) 5°, and (**c**) 10°.

To also capture the influence of different camera angles the camera then was rotated by 5° and 10° around the transversal and longitudinal axis of the prism and the experiment was repeated. All transversal recording sessions are illustrated in Fig. 5 (a) 0°, (b) 5°, and (c) 10°. For the corresponding longitudinal rotation, the prism was rotated by 90° and the process was repeated.

Reconstruction space was only calibrated using the recording of the calibration plate closest to the prism. In this calibration recording a grid of 152 points was marked (a 17 × 9 array of landmarks, omitting one point as a marker for grid orientation). All recordings of the calibration plate further away from the prism were used for 3D reconstruction based on this initial calibration. I.e. these recordings were treated as recordings of “objects for reconstruction”. In these recordings only a 9 × 9 array of landmarks was marked to increase clicking accuracy at a higher distance from the prism, avoiding overlapping markers. Respectively, errors were calculated based on this 9 × 9 grid between 3D reconstructed landmark positions and the known real space landmark positions (ideal positions) at a distance of 1 to 15 mm from the prism.

The effect of six potential influencing factors was investigated: (1) the influence of distance between the prism surface and reconstructed points (2) the influence of rotations on the prism transversal axis (3) the influence of rotations on the prism longitudinal axis (4) the influence of different numbers of points used for calibration in the calibration recording (5) the influence of extrapolation of calibrated space in x- and y direction (6) the influence of changing camera parameters between calibration and reconstruction. The latter was achieved by using the 0° calibration recordings (plate to prism surface distance 0 mm) to calibrate the respective 5° transversal and longitudinal rotation recordings for reconstruction (plate to prism surface distance 1 to 15 mm).

For validation of the 3D reconstruction process, two error measures were computed. **Position error** reflects the exact absolute deviation between each reconstructed 3D grid point position and the expected ideal position of this 3D grid point in real space in x- y- and z-direction. The **Distortion error** relates the measured distances between reconstructed points on the calibration plate to their expected ideal distances i.e. it measures changes in dimensions of the reconstructed grid on the calibration plate compared to known actual dimensions. Distortion of the reconstructed surface is more critical than systematic position misalignment. However, due to the small thickness of the calibration plate distortion can be misleadingly high. For this reason for most evaluations, both error types are included.

### Surface interpolation and cycle detection

**Figure 6:**
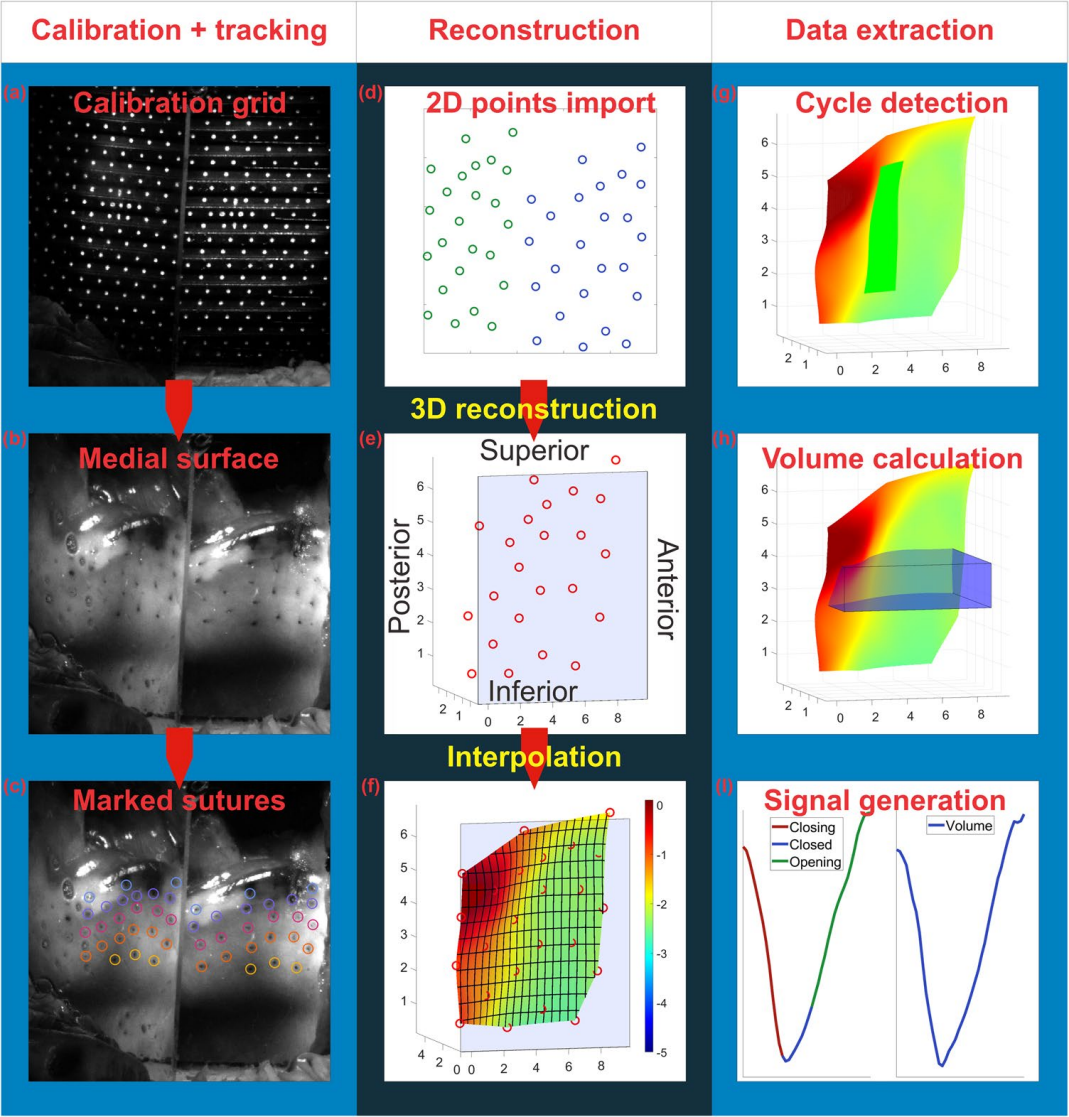
Summary of 3D reconstruction process covering (**a**) calibration, (**b**) medial surface recording, (**c**) tracking of landmarks, (**d**) importing 2D marker positions, (**e**) 3D reconstruction, (**f**) surface interpolation, (**g**) cycle detection, (**h**) volume calculation and (**i**) data generation.

A pictorial summary of the process from in-vivo measurement until 3D data extraction is given in Fig. 6. In the first step (a) a calibration image is recorded. Without changing the camera or prism position after removing the calibration plate (b) the medial surface with an array of tattooed landmarks is recorded. (c) These landmarks are highlighted and tracked. (d) The corresponding 2D positions of all markers are imported in Matlab (If landmarks tattooed on the vocal fold in 2D space were obscured for up to 3 frames in one view or both these positions were linearly interpolated). (e) Using the previously described algorithm the 3D position of each point is reconstructed and (f) the 3D surface shape formed by the 3D point positions was computed by spline interpolation (using Matlab function griddata with interpolation method “cubic”^34^). (g) A narrow inferior-superior slice of the surface, covering 20% of the full anterior–posterior range and 60% of the full superior-inferior range was chosen for cycle detection. (h) Volume was calculated based on a broad surface slice covering 80% of the full anterior–posterior range and 20% of the full superior-inferior range. (i) based on these selections, volume over time and basic phase lengths (opening, closing, and closed) were calculated for demonstration purposes for the three previously mentioned stimulation levels ((1) RLN 4 SLN 5, (2) RLN 5 SLN 5, (3) RLN 6 SLN 5). Measures were calculated individually for three successive cycles and then averaged.

Cycles were determined as follows: Local volume maxima between the narrow surface slice (Fig. 6g) and the prism glass surface determine cycle starts. The closed phase of each cycle is then determined by the minimum distance between the reconstructed surface and glass plate for each reconstructed “line” (i.e. coronal cut) of the surface ranging from inferior to superior in each frame. If more than 50% of the lines that make up a surface slice are closer than 0.02 mm to the prism surface, the corresponding frame is marked as entirely "closed". Afterwards, single unmarked frames that remain between multiple frames marked as “closed” are also marked as closed and vice versa. The closing phase was then determined as ranging from cycle start to the first closed frame, the closed phase as ranging from the second to the second last closed frame, and the opening phase as ranging from the last closed frame to cycle end. All reconstructed videos are included in the Supplementary video collection (Supplementary videos S1).

### Ethics approval

All experiments in this study were carried out complying with the recommendations in the Guide for the Care and Use of Laboratory Animals of the National Institutes of Health. The study was approved by the Animal Research Committee (ARC) of the University of California, Los Angeles (Protocol Number: ARC-2010-021). This study complies with the ARRIVE guidelines.

## Results

### Influence of distance

**Figure 7:**
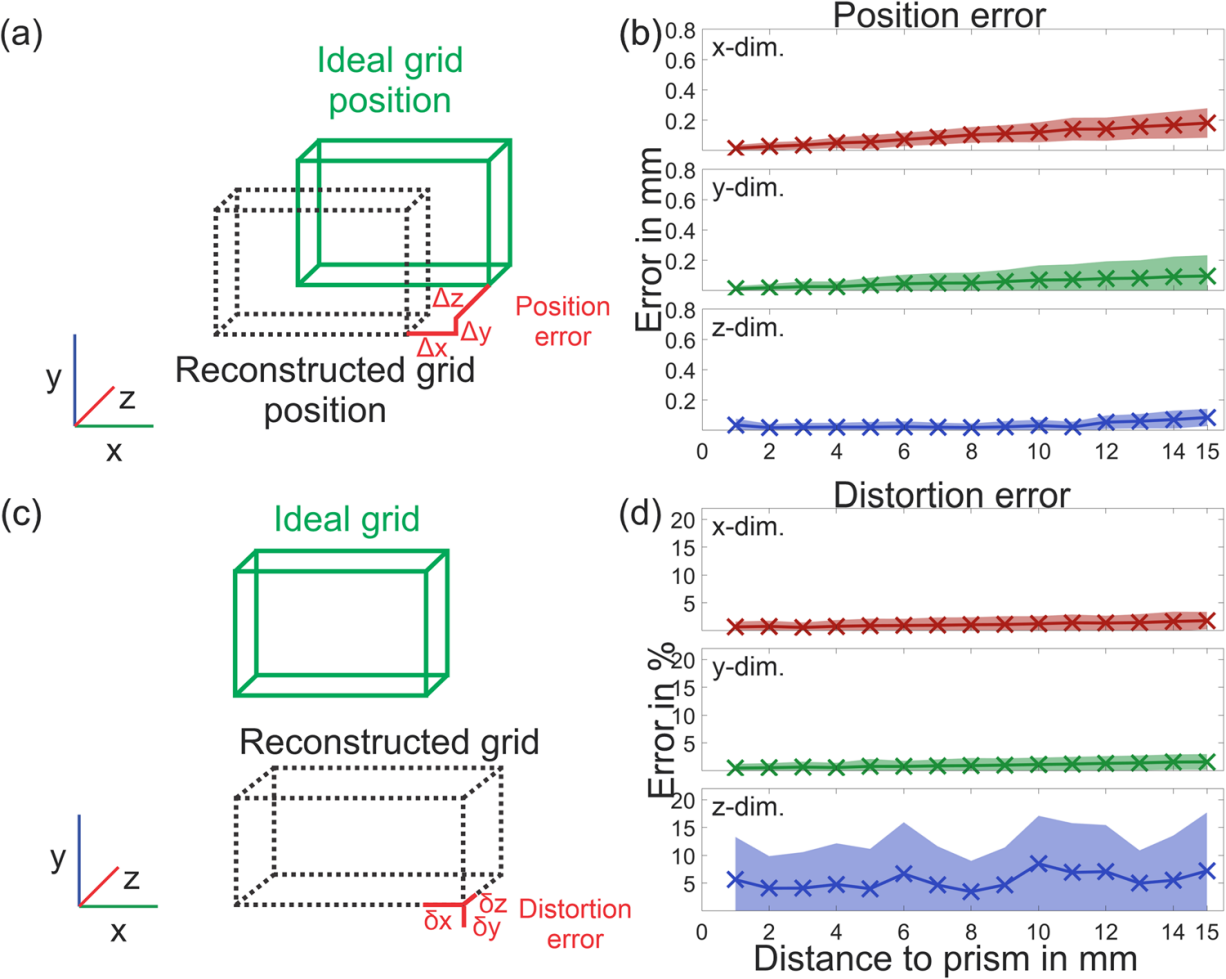
(**a**) Illustration of position error calculation. (**b**) Position error in x-, y- and z-dimension for plate distances from 1 to 15 mm to prism. (**c**) Illustration of distortion error calculation. (**c**) Distortion error in x-, y- and z-dimension for plate distances from 1 to 15 mm to prism.

In Fig. 7 the position error and distortion error for x- y- and z-dimension are depicted for a changing distance to prism from 1 to 15 mm. (a) A schematic visualization of position error and the direction of x-, y-, and z-axis in real space is depicted. Hereby x is defined as the transversal axis of the prism, y its longitudinal axis, and z its frontal axis. (b) The position error in the x-direction and y-direction increases slowly with increasing distance of the plate from the prism. Absolute error in the z-direction shows no noticeable increase with increasing distance. As illustrated in (c) the measured distortion error can be understood as stretching or compression of the reconstructed grid. This error was calculated as a relative value in percent. For (d) x- and y-dimension this error very slightly increases with increasing distance to the prism. The error in the z-direction displays significantly more noise but no unambiguous association with distance to the prism.

### Influence of camera angle

**Figure 8:**
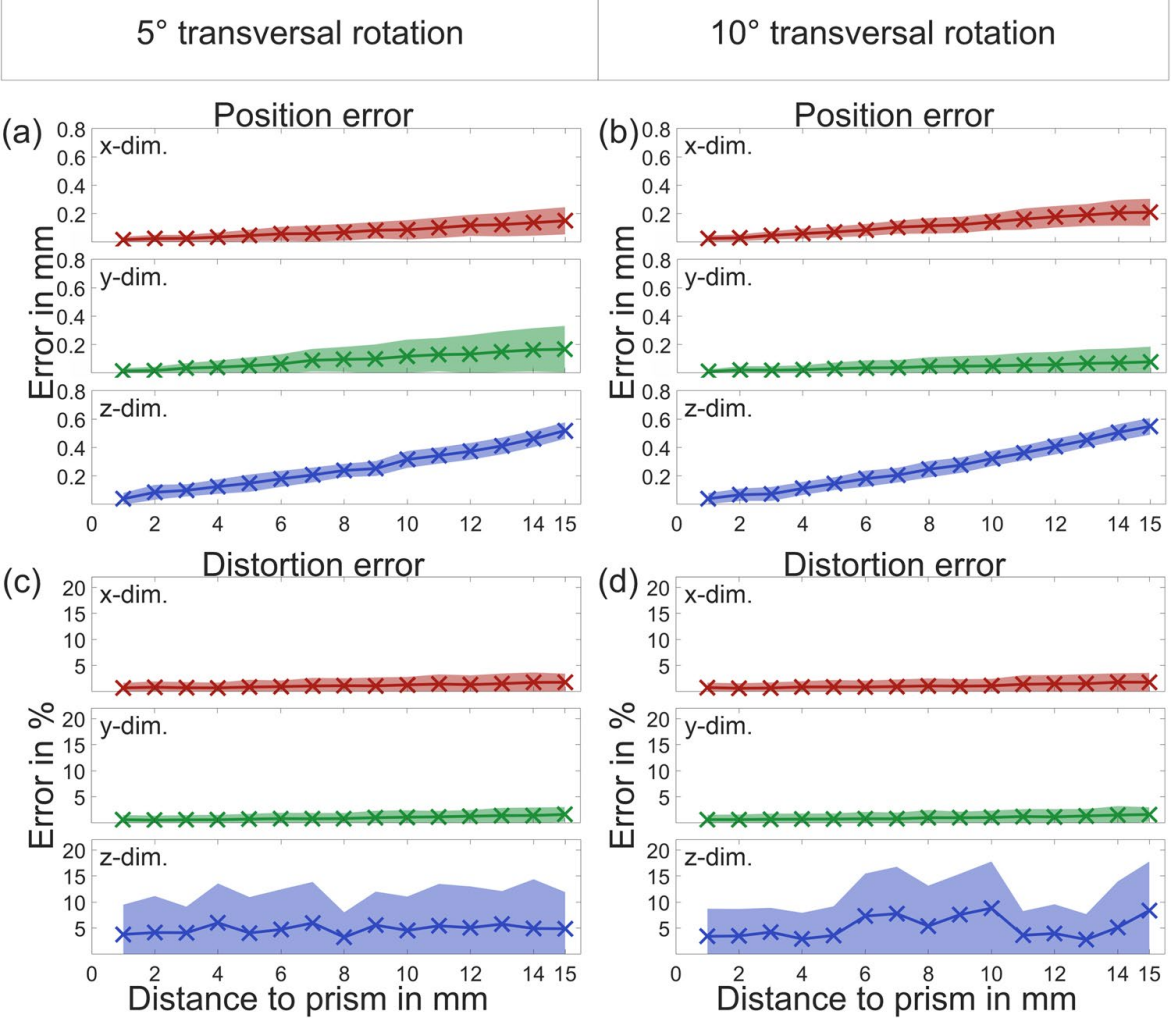
Position error of (**a**) 5° and (**b**) 10° transversally rotated recordings and distortion error of (**c**) 5° and (**d**) 10° longitudinally rotated recordings with two standard deviations.

In Fig. 8 position and distortion errors are shown for 5° and 10° rotations around the transversal axis of the prism. The left column of the figure shows position and distortion errors for 5° rotation and the right column for 10° rotation. As shown in (a) and (b) position error for 5° and 10° stays very low for x- and y-dimension and increases with increasing distance from prism in z-direction. Distortion error for (c) 5° and (d) 10° is only increased in the z-direction.

**Figure 9:**
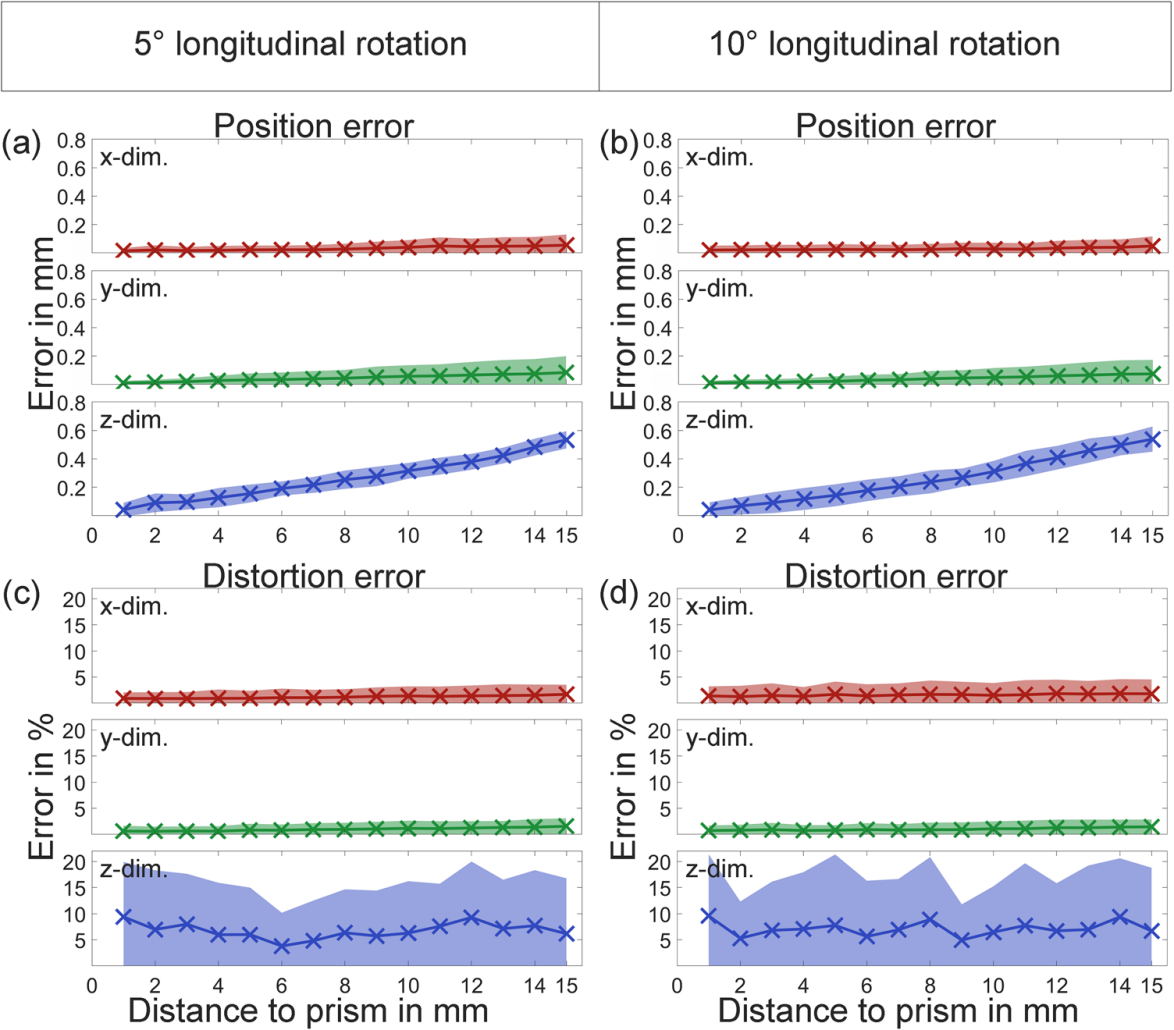
Position error of (**a**) 5° and (**b**) 10° longitudinally rotated recordings and distortion error of (**c**) 5° and (**d**) 10° longitudinally rotated recordings with two standard deviations.

Analogously in In Fig. 9 position and distortion errors are shown for 5° and 10° rotations around the longitudinal axis of the prism. Again position error stays low in the x- and y-direction and increases in the z-direction with increasing distance from prism. Distortion error is also increased in the z-direction.

### Influence of the number of points used in calibration

**Figure 10:**
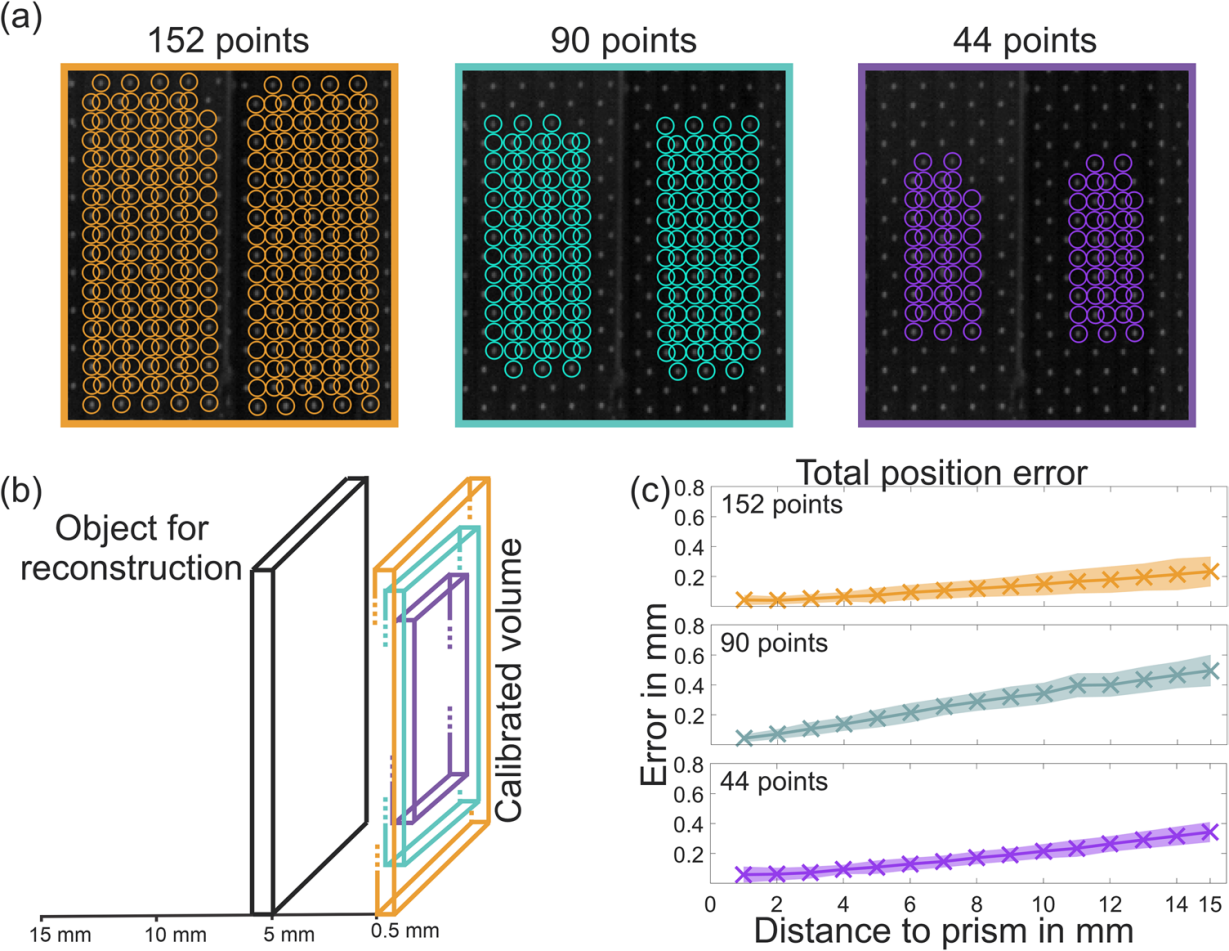
Change in error based on area and number of points used for calibration. (**a**) Images of the three different calibration grids used for this evaluation. (**b**) Schema of the different calibrated areas and where the object for reconstruction would be placed. (**c**) Total position error calculated as the average Euclidean distance between reconstructed points and their ideal positions.

To assess the influence of lower numbers of points used for calibration and smaller calibration volumes (necessitating extrapolation of calibrated space in x- and y-direction) the first evaluation (Influence of distance) was repeated with smaller calibration grids. In Fig. 10a the grids used for calibration are shown from the original 152 points grid and reduced 90 points and 44 points grids. A further reduction to 14 points resulted in the algorithm not converging and is therefore not included here as no error could be calculated. Figure 10b is a schematic illustration of the calibrated volumes and an object for reconstruction relative to calibrated space. Figure 10c shows the total position error for all three calibration grids calculated as the average Euclidean distance between reconstructed points and their ideal positions. The error increases faster with distance in the calibration recordings using a lower number of points. The grid of 90 points had the highest error as for this recording initial calibration error was below the 0.08 mm threshold and hence further optimization was skipped leading to a slightly higher final error. Deformation error (not shown) did not change noticeably between grid sizes.

### Influence of camera parameter change between calibration and reconstruction

**Figure 11:**
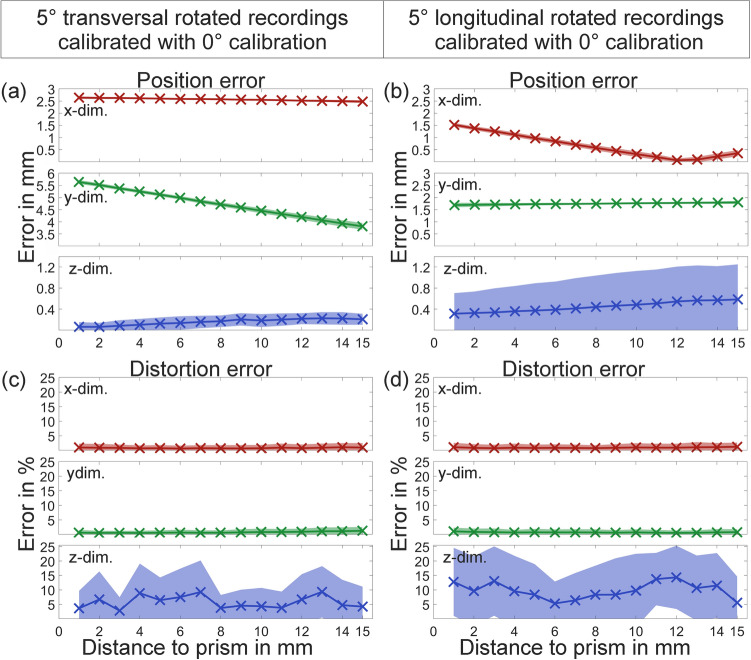
(**a**) position error of 5° transversal rotation recordings calibrated with 0° calibration, (**b**) position error of 5° longitudinal rotation recordings calibrated with 0° calibration, (**c**) distortion error of 5° transversal rotation recordings calibrated with 0° calibration and (**d**) distortion error of 5° longitudinal rotation recordings calibrated with 0° calibration. All errors are given with two standard deviations.

In Fig. 11 position and distortion errors are shown for the experiments exploring the influence of changing camera parameters between calibration and reconstruction. The left column of the figure shows errors if the 5° transversal rotation recordings were calibrated using the 0° calibration recordings, and the right column shows analogously errors if the 5° longitudinal rotation recordings were calibrated using the 0° calibration recordings. As seen in Fig. 11 (a) position error is very high in the x- and y-dimension and moderate in the z-dimension. The error remains largely constant in the x-direction but changes in the y-direction for transversal rotation and (b) vice versa for longitudinal rotation. The nonlinearity in x-dimensional error is due to the reconstructed surface “drifting” in the x-direction with increasing distance from the prism, reaching its ideal position and then moving further in the same direction, moving away from the ideal position again. Also, the standard deviation in the z-dimension is increased. In comparison, the distortion errors as seen in (c) and (d) stay low.

### Comparison with the previous setup

For validation of the previous version of this setup, a calibration error (cE) was calculated based on the distortion of the lengths of the used calibration cube (5 mm^3^). This error was given as an absolute deviation from the ideal cube length of 5 mm for each side and for the cube diagonal^33^. For comparison, we calculated absolute and relative distortion error in the same way based on 2 × 2 × 0.5 grid segments forming cuboids. In Table 1 absolute and relative distortion errors (i.e. deviations in cube/cuboid lengths from their ideal lengths) are given for the previous version of this setup and the current, advanced setup. The absolute error of the advanced setup is between four and 20 times lower.

**Table 1:** Absolute and relative distortion error for the previous and current version of this setup in x, y, and z directions.

Measure	Previous setup	Advanced setup
cE [mm] 0 mm		
cE x-direction	0.13/2.6%	0.01/0.6%
cE y-direction	0.17/3.4%	0.01/0.4%
cE z-direction	0.10/2.0%	0.02/4.8%
cE total	0.24/2.8%	0.03/1.0%
cE [mm] 5 mm		
cE x-direction	0.12/2.4%	0.02/0.9%
cE y-direction	0.08/1.6%	0.02/0.8%
cE z-direction	0.12/2.4%	0.02/4.00%
cE total	0.19/2.2%	0.03/1.1%

### 3D surface reconstruction

In Table 2, average values and standard deviations for the four calculated dynamic laryngeal measures and the three stimulation combinations are given. Additionally, an overall average over conditions and the corresponding standard deviation is given, denoting total variation.

**Table 2:** Average/standard deviation for all stimulation conditions and parameters calculated on three successive cycles. In the last column average/standard deviation of all calculated values for one parameter is given.

Measure	RLN 4 SLN 5	RLN 5 SLN 5	RLN 6 SLN 5	Average
Opening (MS)	5.44/1.58	5.44/0.77	5.11/0.19	5.33/0.85
Closing (MS)	4.00/1.53	3.78/0.19	3.22/0.38	3.67/0.70
Closed (MS)	22.67/1.53	17.56/1.35	18.44/0.51	19.56/1.13
Volume	47.20/12.37	38.39/2.15	36.62/2.86	40.74/5.79

## Discussion

In this study, we adapted the laryngeal 3D reconstruction setup and algorithm originally proposed by Döllinger et al.^31^ in various ways to ease application in in-vivo recordings and improve reconstruction error. We improved feasibility by fixing the prism position and hence circumventing otherwise required continuous re-calibration due to the in vivo animal breathing and hence introducing slight prism movement. We decreased reconstruction error by replacing the calibration cube with a calibration plate, allowing for a larger array of “known points” for calibration and at the same time circumventing most of the otherwise required extrapolation of calibrated space. We added an optimization step to the 3D reconstruction algorithm in case the first F-matrix estimation does not yield convincing results making it more robust to camera angle errors. We performed multiple experiments to quantify reconstruction error based on different potential errors that may happen in real experiments and, lastly, we supplement this work with a Matlab implementation of the discussed algorithm including one example calibration and surface for reconstruction (Supplementary code S2).

As expected, overall error in general increases with increasing distance between the prism surface and reconstructed points. An exception to this trend is position error in z-direction (distance from prism glass plate) that displays a higher dependency from the recording angle and shows no noticeable increase with distance for a perfectly perpendicular angle. In in-vivo settings, a perfectly perpendicular angle cannot always be guaranteed, however, even for the extreme case of 10° camera angle deviation in transversal and longitudinal rotation experiments position z-error does not exceed 0.6 mm at a maximum distance of 15 mm from the prism. Previous hemilarynx phonation studies showed that the vocal fold medial surface in these setups is never farther away than 5 mm from the prism surface, e.g.^35^.

Distortion error describing plate deformation due to reconstruction remains low for x- and y-direction. Distortion error in the z-direction shows high noise but position error is low. This difference is expected as the plate is only 0.5 mm deep in contrast to the much larger expansion in the x- and y-direction (9 mm and 17 mm as covered by marked points). I.e. a distortion error of 10–20% is only equivalent to an absolute deviation of 0.05–0.1 mm.

Lowering the number of points used for calibration, making extrapolation of calibrated space in the x- and y-direction is necessary, only slightly increased overall error. This is expected, as it has been shown before that the linear approximation method used for estimating the F matrix is well-suited for the extrapolation of calibrated space^33^. However, for best results, it may still be advisable to use a large number of points for calibration. The slightly higher error in the 90-point grid is likely due to the omission of the calibration optimization, as the 0.08 mm error threshold was already reached after the first calibration step. The error threshold was set as we noticed that in this error range, additional optimization could result in a lower error in the x- and y-direction at the cost of a higher error in the z-direction.

Calibrating 5° transversal and longitudinal rotation recordings with the 0° calibration recording simulated an accidental small change in camera position between calibration and reconstruction. This resulted in high position errors in x- and y-directions but still acceptable errors in z-direction and low distortion errors. Dependent on rotation, position error in the x- or y-direction changed by approximately 0.5 mm per 5 mm distance from the prism. This implies that this type of change between calibration and reconstruction will mainly lead to changes in the overall position of the reconstructed surface. However, small linear distortions occur depending on the distance from the prism giving the appearance of slightly diagonal movement during vocal fold oscillation, respectively slightly distorting the surface. The higher standard deviation in position error in the z-direction for the 5° longitudinal rotation reconstructions also implies a very slight added rotation of the reconstruction. In the experimental setup, this type of camera parameter change may rarely occur as it can be seen in advance and is usually avoided.

The translational value of this work is in applications where fast-moving objects need to be reconstructed with high precision in a small, accessible 3D domain. One such example is applications in particle image velocimetry (PIV). In tomographic PIV, as particles travel through an air tunnel they pass a laser-illuminated volume that makes particles visible to allow for the 3D reconstruction of their positions in high-speed recordings using four cameras^36^. In setups in which particle overlap is not critical (as particles in the front obscure others behind them), 3D particle positions could be reconstructed using only one camera with the setup validated in this study^37^. Similarly moving or vibrating 3D surfaces of small and medium sizes could be reconstructed when the calibration grid and prism are up- or downscaled accordingly^38^. Further, for translational application, it is critical that sets of paired 2D points can be identified in the split view recordings. This may hinder transferability in some cases. In general, the exact modifications one would have to apply to the described setup and provided source code (Supplementary code S2) depend largely on the specific task that needs to be accomplished and can hence not be described in detail here.

### Summary

As shown by the experiments overall reconstruction error of the proposed procedure is low. Total position error in all directions does not exceed an average of 0.12 mm for the perpendicular camera angle recording at a distance of 5 mm from the prism; the maximum distance the vocal fold surface reaches from the prism in most phonation experiments. Even if considerable camera angle deviations from the perpendicular recording angle are introduced, reconstruction error remains low (c.a. 0.16 to 0.17 mm at a distance of 5 mm from the prism). A reduction in points for calibration necessitating extrapolation in x- and y-directions only slightly increases error as long as a sufficient number of points remain. As long as the vocal fold does not deviate significantly (multiple cm) from the calibrated volume, movement changes, stretching or compression, or changes in distances between landmarks on the vocal fold do not affect the error. What should be avoided are accidental changes in camera position between calibration and reconstruction, but effects are tolerable as long as changes are small. Comparing this setup with its previous version shows a significant improvement in reconstruction error.

### Limitations

Only one canine was used for demonstration and the depth of grooves on the calibration plate is currently only 0.5 mm. Even though the error in z-direction is small, it could likely be further reduced with deeper grooves, allowing for a more accurate calibration in z-direction.

The current 3D reconstruction approach relies on manual marking and tracking of the landmarks on the vocal fold. Due to frequent reflections and shadows as well as limitations of the used camera so far no reliable fully automated tracking algorithm is given. Therefore, the time of marking landmarks is currently the major bottleneck of this 3D reconstruction technique that slows down data generation. We are actively working on improving the process and image quality to allow for the successful introduction of neural network-based automated tracking approaches in the future.

Further, as only the marked points are reconstructed and the remaining surface is interpolated between these points, the accuracy of the 3D reconstruction also depends on the number of points and density of the marked point grid on the vocal fold. However, as the vocal fold has a smooth surface no significant errors should be introduced as long as a sufficient number of points was tattooed.

## Conclusion

In this work, we proposed and validated a new setup for 3D moving tissue surface reconstruction building on previous work from Chhetri et al.^2,3^ and Döllinger et al.^31^. We advanced the 3D reconstruction method by Döllinger et al.^31^ by easing the application for in-vivo recordings by introducing a fixed prism and a plate-based calibration. We added to the reconstruction algorithm making it more robust towards deviations in recording angles and changes in camera parameters. Using the established laryngeal nerve stimulation protocol by Chhetri et al.^2,3^ in combination with these adjustments, in-vivo 3D reconstruction of the oscillating vocal fold medial surface in combination with concurrent nerve stimulation was improved. The precision of this reconstruction was validated and did show a maximal 0.12 mm total position average error at a distance of up to 5 mm from the prism surface for perpendicular recording, outperforming the previous version of this setup^33^ and other established in-vivo 3D reconstruction methods in terms of spatial resolution^23,24^. Lastly, a demonstration of the utility of this setup was given by reconstructing three vocal fold medial surface recordings and calculating basic parameters. The application of this set-up is not limited to voice research, as the algorithms can be applied fully or in part to other problems^37,38^ requiring accurate reconstruction of accessible moving surfaces or particles.

## Data availability

All relevant data for a replication of the described 3D reconstruction algorithm is provided in the supplementary material. For data requests regarding this study please contact pschlegel@ucla.edu.

## Supplementary Information

Supplementary Information 1.

Supplementary Information 2.
